# FERTILITY CARE IN LOW AND MIDDLE INCOME COUNTRIES: Fertility care in low- and middle-income countries

**DOI:** 10.1530/RAF-24-0042

**Published:** 2024-07-04

**Authors:** Willem Ombelet, Federica Lopes

**Affiliations:** 1 The Walking Egg non-profit Organization, Genk, Belgium; 2 Faculty of Medicine and Life Sciences, Hasselt University, Agoralaan, Diepenbeek, Belgium; 3 School of Medicine, University of Dundee, Dundee, United Kingdom

**Keywords:** fertility care, low- and middle-income countries

## Abstract

Infertility affects millions worldwide, with significant medical, financial, and emotional challenges, particularly in low- and middle-income countries (LMICs). Cultural, religious, financial, and gender-related barriers hinder access to treatment, exacerbating social and economic consequences, especially for women. Despite its prevalence, infertility often remains overlooked due to competing health priorities. However, global initiatives recognise infertility as a reproductive health concern, advocating for universal access to high-quality fertility care. In LMICs, limited resources and infrastructure impede access to treatment, prompting people to turn to alternative, often ineffective, non-biomedical solutions. Addressing these challenges requires implementing affordable fertility care services tailored to local contexts, supported by political commitment and community engagement. Emerging technologies offer promising solutions, but comprehensive education and training programs are essential for their effective implementation. By integrating fertility care into broader health policies and fostering partnerships, we can ensure equitable access to infertility treatment and support reproductive health worldwide.

## Introduction

Infertility is one of the most common chronic diseases among people of childbearing age, affecting roughly 8% to 12% of reproductive-aged couples worldwide (Boivin *et al.* 2007), with men and women contributing almost equally to the overall cases. Depending on the criteria used to assess it, the worldwide prevalence of involuntary childlessness varies between 52.6 and 200 million couples, the majority being residents of low- and middle-income countries (LMICs) (Rutstein & Iqbal 2004, Ombelet *et al.* 2008, Mascarenhas *et al.* 2012).

Infertility poses a number of medical, financial, and emotional challenges in high-income countries, and these are drastically amplified in LMICs. Lack of infrastructure, prohibitive costs, and cultural and religious barriers are among the most common reasons for reduced access to medically assisted reproduction (MAR) in many parts of the world. In addition, in most LMICs, infertility and childlessness are gender inequality issues, and the burden of the problem lies predominantly on the female partner. In many of these communities, women are stigmatised, ostracised, and become targets of psychological abuse or intimate partner violence, even if infertility is due to the male partner (Dyer *et al.* 2004, Ombelet *et al.* 2008, Inhorn & Patrizio 2015, Wang *et al.* 2022).

On the other hand, the most important obstacle to implementing health policies that address infertility in LMICs is the widespread belief that infertility is not a pressing problem in countries where fatal and contagious diseases remain uncontrolled. Because infertility itself is not immediately life-threatening, it remains a low priority for local health care providers and community leaders, despite the high prevalence of infertility and the severe social and economic consequences of childlessness in LMICs (Ombelet 2008, Chiware *et al.* 2021).

As confirmed by the 2018 report of the Guttmacher-*Lancet* Commission on Sexual and Reproductive Health and Rights (SRHR), infertility is a reproductive health concern that deserves attention (Starrs *et al.* 2018). In 2020, the World Health Organization (WHO) published a fact sheet on infertility, and the message was clear: infertility is recognised as a disease, and universal access to high-quality services for family planning, including fertility care, is one of the core elements of reproductive health (WHO 2020). With this fact sheet, the WHO acknowledged that patients have a right to treatment and highlighted the urgent need for accessible and affordable infertility care worldwide, including in LMICs. Although not an immediate danger to physical health or threat to life, infertility has been ranked as the fifth highest cause of global disability due to its significant implications on the biopsychosocial well-being of individuals (ESHRE Factsheet 2021). One of the targets of the United Nations Sustainable Development Goal 3 is to ensure universal access to sexual and reproductive healthcare services by 2030.

On the other hand, the actual situation concerning infertility in LMICs shows that many shortcomings can be identified insofar as infertility remains an important medical problem in almost all LMICs due to unsuccessful or non-existent educational and/or prevention programmes, inaccessible or unavailable diagnostic procedures, and a great shortage of relevant treatment options.

This series will explore some of the challenges and barriers to accessing infertility care in different critical LMICs, with articles from global experts in the field. The series will cover original articles to increase awareness of this crucial medical and scientific topic, promote infertility treatment in the agenda of policymakers and funding bodies, and explore new avenues for the development of affordable fertility care in LMICs.

*Reproduction and Fertility* is an open access journal and as such, is accessible to the wide scientific community, ensuring that this series makes a significant impact in the field.

## Socioeconomic and cultural aspects of infertility in LMICs

Particularly in countries where childbearing is highly valued, infertility is not only a reproductive health issue but also a socio-economic issue that can affect marital, family, and other interpersonal relationships. Because many families in LMICs are completely dependent on children for economic survival, childlessness must be seen as a social and public health issue, not just an individual’s medical problem. Even without the economic burden, involuntary childlessness is often associated with devastating psychological and social consequences.

Infertility is associated with poor mental health, social isolation, loss of social status, relationship dissolution, and catastrophic expenditure on treatment (Dyer & Patel 2012, Inhorn & Patrizio 2015, Asiimwe *et al.* 2022). This can lead to stigma, ostracism, anxiety, depression, and low self-esteem. Women in LMICs are particularly vulnerable to the negative consequences of childlessness. As a result of a systematic review and meta-analysis, Wang *et al.* (2022) concluded that at least one in three infertile women in LMICs experienced intimate partner violence (IPV) over a 12-month period and about one in two over their lifetime. Psychological violence was found to be the most common form of IPV, followed by physical violence, sexual violence, and economic coercion.

## Religion and assisted reproduction in LMICs

Human response to new developments in birth and death is largely influenced by religious beliefs. The introduction of assisted reproductive technologies (ART) into medical practice in the last quarter of the 20th century was fiercely attacked by some religious groups, while warmly welcomed by others. The birth of the first IVF baby in 1978 presented the world with the sobering fact of the possibility of achieving pregnancy and birth through methods previously unthinkable. Like many overwhelming achievements, many people passed through the stages of denial, confusion, and finally acceptance (Sallam & Sallam 2016).

Culture and religion were and are among the key stakeholders in MAR, if not the stumbling blocks to acceptance of ART, with religious figures in some countries dictating what procedures are allowed and what cannot be performed. (Serour & Serour 2021). This is mostly due to the different cultural and religious perspectives on the moral status of the embryo, fundamental beliefs related to family and inheritance, and concerns about what could be done with human embryos in the laboratory. However, with over eight million children being born with MAR, there is increasing pressure to combine and integrate moral and religious beliefs with the growing need for MAR worldwide and its more widespread social acceptance; yet, this is far from easy to achieve. Religion will always play a major role in people’s attitudes towards ART, and the debate is sure to continue as new developments emerge in the constantly evolving field of assisted reproduction.

## Access to infertility care in LMICs

Despite the need for comprehensive infertility care and increasing demand for services, access to infertility treatment in LMICs, particularly in sub-Saharan Africa (SSA) remains very limited or unavailable for most infertile couples and individuals (Ombelet *et al.* 2008, Gerrits & Shaw 2010). Untreated sexually transmitted infections (STIs) are a leading cause of female and male infertility due to inadequate diagnostic services and disparities in treatment access. Other major causes of female infertility, such as unsafe abortion and childbirth complications, require more advanced treatment and better handling by reproductive health care providers (Ombelet 2008). Published data indicate that less than 1.5% of the population in SSA has access to MAR (Ombelet & Onofre 2019).

Among several barriers to improving access to infertility services in SSA, cost is the major barrier, with more advanced diagnostic and treatment services primarily provided by the private sector (Afferri *et al.* 2022, Asiimwe *et al.* 2022), with fees often being prohibitive for the general population. Extremely small public resources are directed to MAR, and even when infertility care is available in public facilities, it is often poorly coordinated, with high out-of-pocket costs, and does not always offer complex services and more advanced techniques (Gerrits & Shaw 2010, Majangara Karaga *et al.* 2023).

Other logistical and cultural barriers to accessing infertility treatment are also important, such as geographical barriers, being in a non-traditional relationship, and the role of the male partner in preventing or discouraging treatment (Gerrits & Shaw 2010, Afferri *et al.* 2022). As a consequence of this, infertile couples and individuals may seek non-biomedical sources of fertility treatment. Traditional and religious healers play an important role and are a popular alternative to biomedical care in SSA, offering counseling, coping strategies, and medicines that are often more affordable and culturally appropriate. For those who do seek care from the formal health system, it is often regarded as a last resort after traditional methods have failed. As a consequence, many infertile couples and individuals wait too long to seek medical treatment, which greatly reduces their chances of a successful outcome. Ultimately, for people struggling to conceive in many LMICs, the source of infertility care they seek is not motivated by preference, but rather by local availability, affordability, and accessibility of services, and may be the last choice after traditional treatments, which are mostly ineffective and may even be harmful, delaying access to more effective treatments and not without significant financial cost.

## Implementation of affordable infertility care services in LMICs

The implementation of accessible, low-cost fertility clinics in LMICs, with affordable, effective, safe, and standardised diagnostic and therapeutic procedures has to be regarded as a priority to increase accessibility to high-quality infertility care. When setting up low-cost infertility care services, country-specific socio-cultural and religious/moral notions, gender relationships, and local (economic) circumstances should be taken into account. The integration of infertility management into sexual and reproductive health care programmes and a reduction of costs are considered prerequisites for implementing “new reproductive technologies” in LMICs. Simplifying ART procedures and minimising complication rates will be mandatory if we want to make MAR available and accessible, especially outside the private health care sector. Ideally, these infertility services should be implemented into existing reproductive health care programmes with special interest in family planning and mother care (Ombelet *et al.* 2008). Until now, infertility care in LMICs is almost 100% linked to the private sector and therefore unreachable for the large majority of the population. Due to the high cost of setting up an ART unit, especially the laboratory section, careful financial planning is required, taking a myriad of factors into consideration.

As LMICs differ in their stages of development, three levels of implementation are suggested:

**Level 1** – A basic infertility clinic capable of offering the following services: basic infertility workup including semen analysis, hormonal assays, follicular scanning, ovulation induction, and IUI (intrauterine inseminations).

**Level 2** – An advanced infertility clinic capable of offering the following services in addition to level 1: IVF, diagnostic endoscopy.

**Level 3** – A tertiary level infertility clinic capable of offering the following services in addition to level 2: ICSI, cryopreservation, operative endoscopy, etc.

Another possible model to increase the number of ART centres, without the financial burden of setting up an ART laboratory for each centre, is a single laboratory that can provide a service to multiple facilities through the establishment of a mobile ART laboratory.

Effective implementation of these initiatives requires (1) sustained political support, (2) public sensitisation and engagement of traditional, cultural, and religious leaders, (3) strengthening local innovation and capacity building of fertility health workers, and (4) proven clinical evidence and utilisation of cost-effective initiatives in LMICs.

A major barrier holding back the successful setting up of ART centres is the critical shortage of medical doctors, nurses, social workers, embryologists, and laboratory technicians with some familiarity with reproductive medicine, especially in SSA. The scarcity of well-trained, locally available embryologists is commonplace in Africa although the role of an embryologist in an IVF laboratory is indispensable and of major importance. Even many private clinics, through transnational networking, opt for using qualified embryologists coming from Europe or India for a short time to carry out the laboratory procedures.

New technologies may also provide alternative avenues for democratising infertility care. A number of highly automated instruments are under development (Abdullah *et al.* 2023), promising to increase the throughput of laboratory processes that are currently labour- and time-consuming. Sperm and oocyte selection tools (Mendizabal-Ruiz *et al.* 2022), embryo grading machines (Salih *et al.* 2023), robotic ICSI rigs, and other equipment (Nauber *et al.* 2023) are taking advantage of the fast developments in artificial intelligence. Although these technological advancements are unlikely to replace humans, they may revolutionise the future role of embryologists, reproductive biologists, and data analyst experts, while increasing efficiency and improving the use of resources. Furthermore, the development of home testing phone apps may allow some of the basic tests to be performed by patients without the need for time-consuming and costly clinic visits (Onofre *et al.* 2021). Therefore, we believe that the implementation of new reproductive technologies in LMICs will require well-organised educational and training programmes. Training courses should be tailored to the local conditions and the possible difficulties encountered in LMICs. Following training, quality control, regular audit, and systems of accreditation and registration should be implemented by each country in order to maintain appropriate standards of care.

In LMICs, the integration of fertility care into reproductive health policy is still fragmented or does not exist at all. It will depend upon factors including the recognition of infertility as a disease, as well as strong political, social, and religious engagement. Up to now, international foundations, NGOs, charities, local politicians, insurance companies, and health care providers are not interested in funding infertility care because ART is still associated with very high direct and indirect costs. To develop a brighter future for MAR in LMICs, a proactive approach seeking more affordable strategies to deliver reproductive care, implementing cost-effective initiatives, and developing new training programmes through public-private, local, and international partnerships will be crucial.

## Declaration of interest

The authors declare that there is no conflict of interest that could be perceived as prejudicing the impartiality of this commentary.

## Funding

This work did not receive any specific grant from any funding agency in the public, commercial, or not-for-profit sector.
